# Development of a prognostic model for patients with extensive-stage small cell lung cancer undergoing immunotherapy and chemotherapy

**DOI:** 10.3389/fimmu.2025.1561333

**Published:** 2025-03-07

**Authors:** Yunbin Gao, Lixia Zhang, Meng Yan, Zongwen Sun, Haibo Zhao, Lujun Zhao

**Affiliations:** ^1^ Department of Radiation Oncology, Tianjin Medical University Cancer Institute and Hospital, National Clinical Research Center for Cancer, Tianjin Key Laboratory of Cancer Prevention and Therapy, Tianjin’s Clinical Research Center for Cancer, Tianjin, China; ^2^ Department of Oncology, Jining No. 1 People’s Hospital, Jining, Shandong, China

**Keywords:** immunotherapy, chemotherapy, prognosis, nomogram, extensive-stage small cell lung cancer

## Abstract

**Purpose:**

In this study, we aimed to develop a predictive model for patients receiving chemotherapy and immunotherapy for extensive-stage small cell lung cancer

**Methods:**

We retrospectively analyzed 112 extensive-stage small cell lung cancer patients treated with first-line immunotherapy and chemotherapy. The relevant clinical data were collected to evaluate the changes during the treatment. The best subset regression, univariate analysis, and LASSO regression with cross-validation were applied for variable selection and model establishment. The nomograms for 1- and 2-year survival probabilities were established, and the calibration curve was utilized to evaluate the correspondence between actual and predicted survival. The model prediction capacity was assessed using decision curve analysis, calibration curves, and receiver operating characteristic curves. Moreover, five-fold cross-validation was conducted for internal validation. According to risk score, the patients were assigned to high- and low-risk groups, and survival curves were generated for each group.

**Results:**

The LASSO regression model was established based on the variables such as age, ECOG, metastatic sites, NLR, and immunotherapy cycles. This predictive model displayed robust performance, evidenced by the Area Under the Curve of 0.887 and concordance index of 0.759. The nomogram effectively predicted 1- and 2-year survival probabilities and demonstrated a high degree of calibration. The decision curve analysis displayed that the model possessed superior predictive capability. The risk stratification for patients with high- and low-risk categories facilitated more individualized survival assessment.

**Conclusion:**

The study successfully developed a prognostic model for extensive-stage small cell lung cancer patients undergoing immunotherapy and chemotherapy, demonstrating the good accuracy and predictability.

## 1 Introduction

Approximately 15% of worldwide lung cancer cases are small cell lung cancer (SCLC), exhibiting a high degree of invasiveness and rapid progression (1, 2). It is distinguished by its poor prognosis and early metastatic potential. According to the feasibility of covering an irradiation field, SCLC patients are frequently categorized into limited-stage disease and extensive-stage disease. Approximately 70% of all SCLC cases are classified as extensive-stage (3). For extensive-stage SCLC (ES-SCLC) patients, first-line platinum-based chemotherapy regimens have served as the standard treatment option. Over the past 20 years, the median overall survival (mOS) for patients with ES-SCLC following conventional treatment has maintained between 9 and 11 months (4). Immunotherapy has revolutionized the cancer treatment paradigm and has shown promising activity in numerous solid malignancies, including SCLC. The results of CASPIAN and IMpower133 trials suggested that the combination of chemotherapy and immunotherapy improved mOS by two months compared to chemotherapy alone in ES-SCLC patients (5, 6). As a result, according to the National Comprehensive Cancer Network (NCCN) guidelines, platinum-based chemotherapy with etoposide and durvalumab or atezolizumab was considered as the standard first-line treatment for ES-SCLC (7).

Only a few biomarkers, such as programmed death-ligand 1 (PD-L1) immuno-histochemistry (IHC) expression, tumor mutational burden (TMB), and circulating tumor DNA (ctDNA), have been investigated in relation to immunotherapy to predict the clinical prognosis of SCLC (3, 8, 9).

The principal objective of this study is to develop a predictive model for assessing the response of patients with ES-SCLC to initial chemotherapy and immunotherapy treatments. The proposed conceptual model aims to aid healthcare professionals in devising personalized treatment strategies by identifying potential biomarkers and clinical predictors tailored to the individual needs of each patient. This approach has the potential to enhance therapeutic outcomes and improve the overall prognosis of the disease.

## 2 Materials and methods

### 2.1 Patients

The study participants included patients diagnosed with ES-SCLC at Tianjin Medical University Cancer Institute between 2018 and 2022. Inclusion criteria: (1) patients had to be 18 years and above; (2) diagnosis with an extensive stage based on the Veterans Administration Lung Cancer Group (VALG) staging criteria; (3) Eastern Cooperative Oncology Group (ECOG) performance status of 0-2; (4) receipt of chemotherapy and immunotherapy as their initial treatment. Exclusion criteria: (1) prior neoplastic or autoimmune disease. (2) receipt of anti-tumor treatment in the past, including chemotherapy, radiotherapy, and immunotherapy. This investigation was authorized by the Institutional Review Board (IRB) of Tianjin Medical University Cancer Institute and Hospital (approval number: bc2022244).

### 2.2 Data collection

Collection of clinical data in this study included ECOG performance status (PS), age, smoking history, gender, co-morbidities (such as hypertension, diabetes, and coronary heart disease), a familial predisposition to lung cancer, weight loss, disease stage, tumor localization (specifically left or right lung and lung lobe location), presence of oligometastases, metastatic sites (including brain, bone, liver, adrenal gland, abdomen, pleural fluid, and pleural nodules), neutrophil-to-lymphocyte ratio (NLR), monocyte-to-lymphocyte ratio (MLR), lactate dehydrogenase (LDH) levels, albumin (ALB) levels, platelet-to-lymphocyte ratio (PLR), number of immunotherapy cycles, systemic immune-inflammation index (SII), and the response to first-line immunotherapy. Overall survival (OS) was the primary endpoint, defined as the time interval between initiation of immunotherapy and death or the final follow-up date.

### 2.3 Model construction and validation

Variable selection was performed using three methods: Simple regression analysis, best subset regression analysis, and LASSO regression analysis using cross-validation techniques. All the selected variables were employed to develop a survival probability model for survival rates of 1 and 2 years. In order to measure the performance of the model, three tools were used: the calibration curve, the decision curve analysis (DCA), and the receiver operating characteristic (ROC) curve analysis. After analyzing the model’s performance based on the ROC curve using the variables selected via three distinct approaches, the best model was determined according to the maximum area under the curve (AUC). The outcomes for patients were evaluated using the nomogram. The decision, calibration, and ROC curves were utilized for assessing the model’s discriminative capacity. The internal validation was performed utilizing cross-validation presented as scatter plot. All patients were rated using the previously indicated model, and the R software was utilized to identify the optimal cutoff value. The final model classified patients into high- or low-risk categories. To compare the survival probabilities for these groups, the Kaplan-Meier curve was plotted as well.

### 2.4 Statistical analysis

R version 4. 1. 3 was utilized to perform the statistical analysis in this study. *P* value less than 0.05 is considered to be statistically significant. By dividing the medians using Kaplan-Meier, the variables, which included LDH, ALB, NLR, PLR, MLR, and SII, were stratified into high- and low-risk groups. These estimates comprised survival rates at one and two years and the corresponding 95% CI, produced using the Kaplan–Meier analysis.

## 3 Results

### 3.1 Baseline characteristics

This research examined 112 individuals with ES-SCLC. The median age of the participants was 63 years (IQR 57.7, 68.0), with 81.2% of them being male. An ECOG PS score of 1 (n = 102, 91.1%) was obtained for the majority of patients. As shown in **Table 1**, the baseline characteristics of these participants were summarized. By the date of last follow-up, 54 patients died, 24 patients were alive with disease progression, and 34 patients were alive without disease progression. A median progression-free survival (PFS) of 4.7 months (95% CI, 4.0-5.7) and an overall survival (OS) of 12.1 months (95% CI, 10.4-15.4) were reported. The OS rates at 1 and 2 years were 60.1% and 29.4%, respectively.

**Table 1:** Baseline patient characteristics.

Characteristic	Patients (%)
Total	112
Sex	
Female	21 (18.8)
Male	91 (81.2)
Age (median [IQR])	63.0 [57.75, 68.0]
Age (≥65)	
No	65 (58.0)
Yes	47 (42.0)
ECOG PS	
0	3 (2.7)
1	102 (91.1)
2	7 (6.2)
Smoking (%)	
No	20 (17.9)
Yes	92 (82.1)
Basic disease	
No	56 (50.0)
Yes	56 (50.0)
Family history of cancer	
No	86 (76.8)
Yes	26 (23.2)
Lose weight	
No	97 (86.6)
Yes	15 (13.4)
T	
T1	14 (12.5)
T2	29 (25.9)
T3	27 (24.1)
T4	42 (37.5)
N	
N1	6 (5.4)
N2	44 (39.3)
N3	62 (55.4)
M	
M0	3 (2.7)
M1a	21 (18.8)
M1b	10 (8.9)
M1c	78 (69.6)
Stage	
IIIB	2 (1.8)
IIIC	1 (0.9)
IVA	29 (25.9)
IVB	80 (71.4)
Oligo metastases	
No	38 (33.9)
Yes	74 (66.1)
Brain metastases	
No	87 (77.7)
Yes	25 (22.3)
Bone metastases	
No	61 (54.5)
Yes	51 (45.5)
Hepatic metastases	
No	86 (76.8)
Yes	26 (23.2)
Adrenal metastases	
No	95 (84.8)
Yes	17 (15.2)
Abdomen metastases	
No	99 (88.4)
Yes	13 (11.6)
Hydrothorax metastases	
No	76 (67.9)
Yes	36 (32.1)
Pleura involvement	
No	96 (85.7)
Yes	16 (14.3)
Lung site	
Left	50 (44.6)
Right	62 (55.4)
Lobe site	
upper	69 (61.6)
middle	2 (1.8)
lower	41 (36.6)
LDH	
Low	86 (76.8)
High	26 (23.2)
ALB	
Low	88 (78.6)
High	24 (21.4)
NLR	
Low	21 (18.8)
High	91 (81.2)
PLR	
Low	10 (8.9)
High	102 (91.1)
MLR	
Low	18 (16.1)
High	94 (83.9)
SII	
Low	13 (11.6)
High	99 (88.4)
Immunotherapy cycles (median [IQR])	6.0 [4.0, 10.0]
Response to first-line immunotherapy	
PD	42 (37.5)
PR	34 (30.4)
SD	36 (32.1)

ECOG PS, Eastern Cooperative Oncology Group Performance Status; LDH, Lactate dehydrogenase; ALB, Albumin; NLR, Neutrophil to lymphocyte ratio; PLR, Platelet to lymphocyte ratio; MLR, Monocyte to lymphocyte ratio; SII, Systemic immune inflammation index; PD, Progressive disease; PR, Partial response; SD, Stable disease.

### 3.2 Variable screening

**Figure 1:**
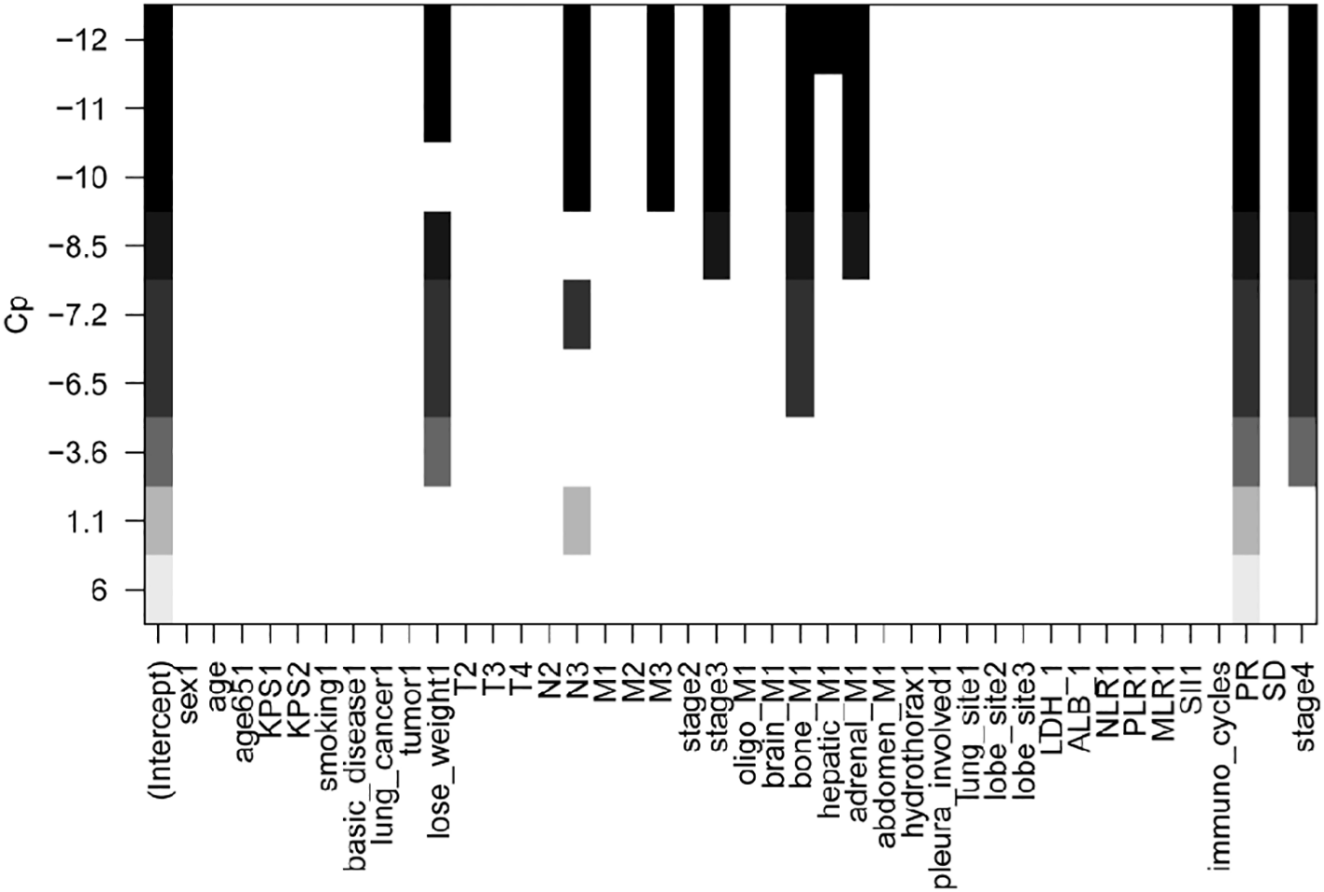
Best subset regression results.

Three methods were used for variable screening: univariate analysis, optimal subset regression, and LASSO regression with cross-validation. All significant variables had a *p*-value < 0.05 in the univariate analysis. Age, LDH, NLR, SII, hepatic metastases, and the number of immunotherapy rounds were among the significant variables. The best subset regression model was determined based on specific criteria, which included a minimum CP value of 12, a maximum adjusted R2 value of 0.3, and a minimum Bayesian Information Criterion value of -6.1. Nine variables were shown to be the most effective combination (**Figure 1**). These were weight loss, N3, M3, stage 3, stage 4, bone metastasis, hepatic metastasis, adrenal metastasis, and partial response (PR).

**Figure 2:**
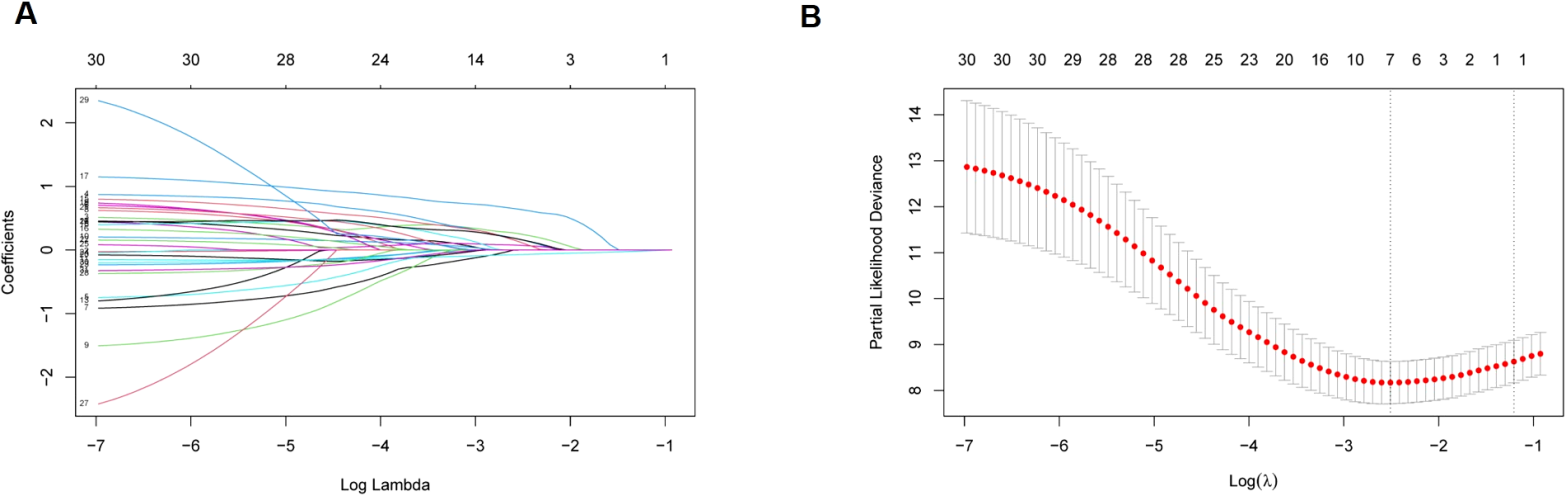
Lasso regression analysis. **(A)** Coefficient path diagram; **(B)** Cross-validation curve.

**Figure 3:**
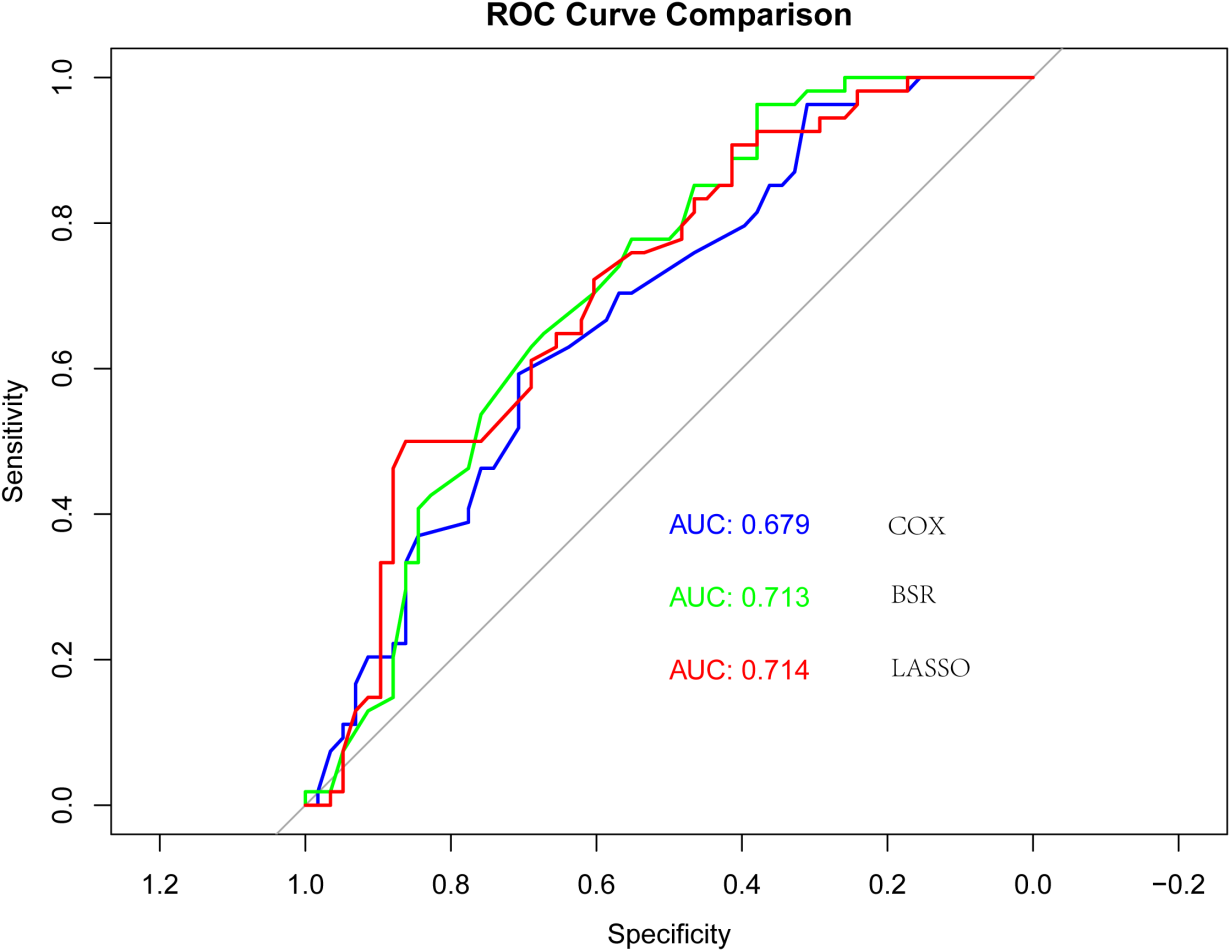
Receiver operating characteristic (ROC) curves for the three models: “COX”, “BSR” and “Lasso”.

A model was constructed through Lasso regression, with the initial variables comprising elderly, ECOG PS, brain metastases, hepatic metastasis, NLR, and immunotherapy cycles (**Figure 2**). The Akaike’s Information Criteria (AIC) values of the three models were compared using COX regression, and the ROC curve analysis was utilized for assessing the models’ performance. The model with the highest AUC value and the lowest AIC was deemed optimal (**Figure 3**). Furthermore, the variables identified through LASSO regression screening were used to construct the nomogram.

### 3.3 Construction of the prognostic model

**Figure 4:**
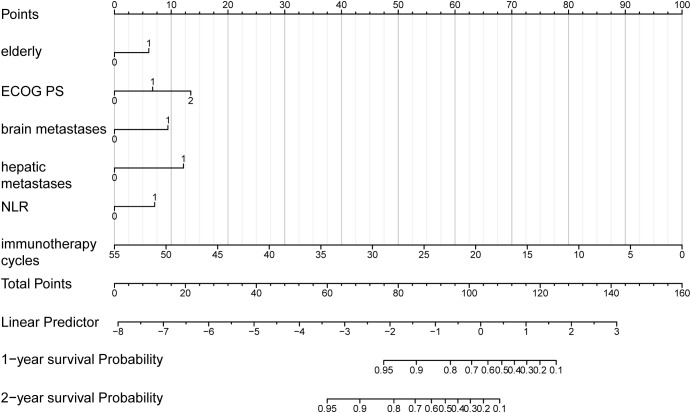
Nomogram for the predictive model for survival in ES-SCLC patients undergoing immunotherapy and chemotherapy (PMSSIC).

To determine the probability of survival at one and two years, a nomogram was generated using the variables previously mentioned (**Figure 4**). The nomogram was titled “Predictive Model for Survival in Extensive-Stage SCLC Patients Undergoing Immunotherapy and Chemotherapy” (PMSSIC). Furthermore, an online tool was created based on the PMSSIC framework for the efficient operation of individual clinical predictions. The tool can be accessed at: https://gaoyunbinjiayou.shinyapps.io/DynNomapp/.

### 3.4 Model evaluation and validation

**Figure 5:**
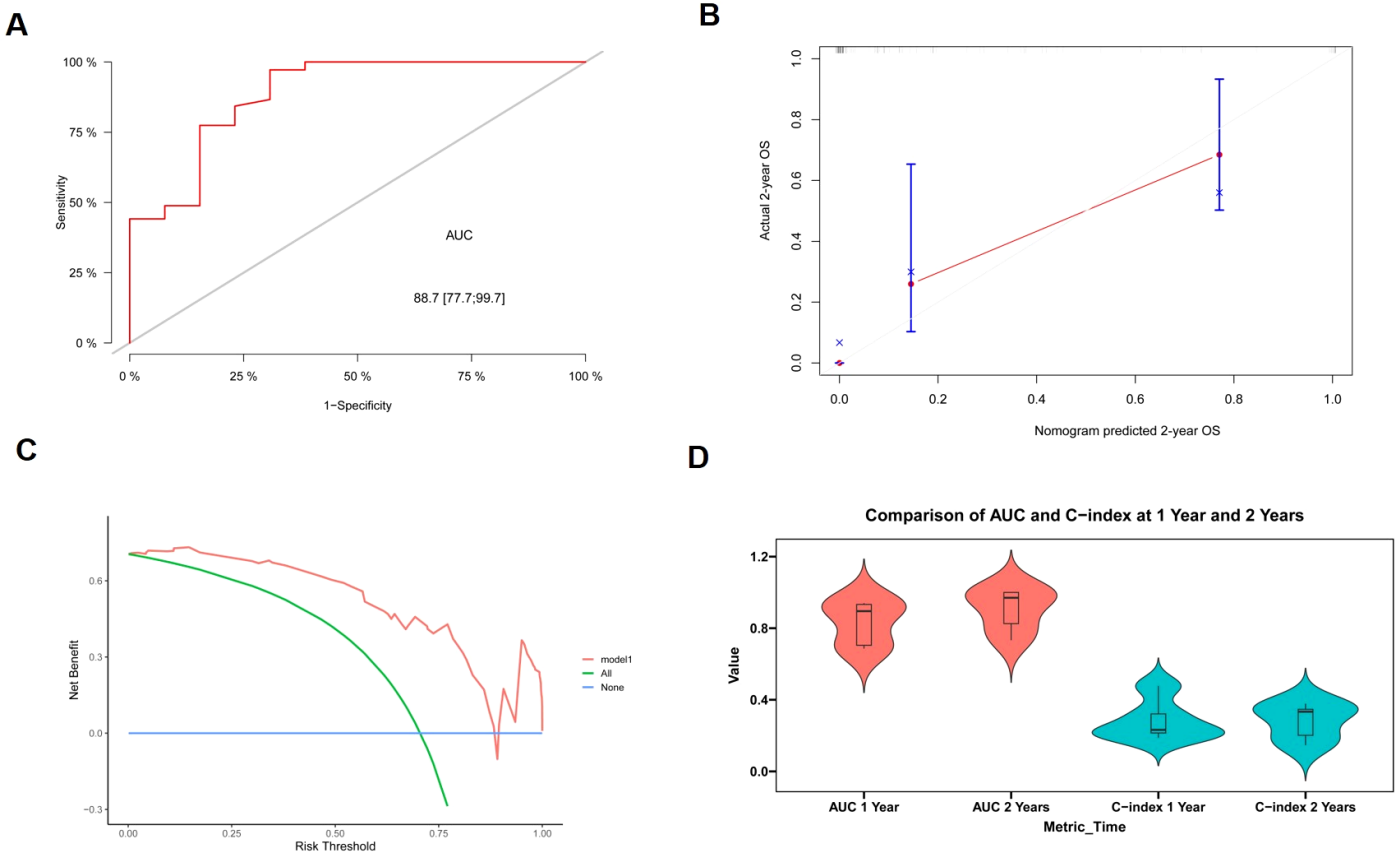
The evaluation and validation of the model. **(A)** Receiver operating curve for model prediction of 2-year survival. **(B)** Calibration curve for model prediction of 2-year survival. **(C)** Decision curve analysis for model prediction of 2-year survival. **(D)** Violin plot depicting the area under the curve (AUC) and C-index for 1- and 2-year intervals.

ROC analysis, calibration plots, and DCA were used to evaluate the nomogram’s stability (**Figures 5A–C**). This model’s 2-year survival probability showed that it had a robust discriminant power, as evidenced by the AUC value of 0.887 (with variance 0.777-0.997). DCA, which demonstrated a positive net benefit based on the probability of 2-year survival on the application of the model, was used to show the utility of the design. The model’s performance was further corroborated by internal cross-validation results, which show mean AUC values at the 1- and 2-year of 0.832 (range 0.686-0.940) and 0.906 (range 0.733-1.000), respectively (**Figure 5D**).

### 3.5 Risk stratification

**Figure 6:**
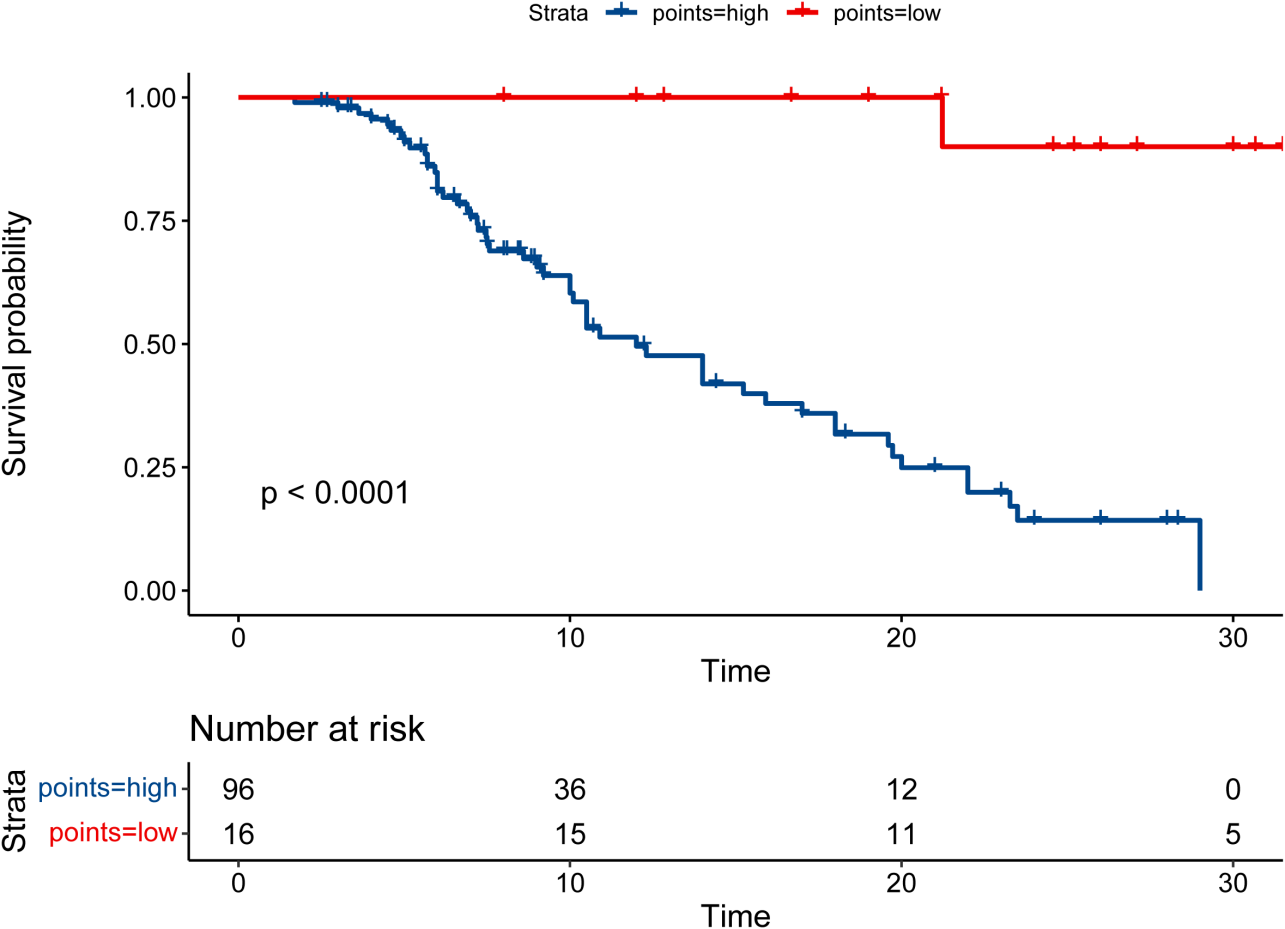
The Kaplan-Meier survival curves for overall survival (OS) in the high- and low-risk groups.

Therefore, the patients were allocated to high- and low-risk groups based on the RS that PMSSIC determined. Moreover, the result of Kaplan-Meier survival analysis indicated that the survival rate of the patients in low-risk group was significantly longer than that of high-risk group (*P* < 0.001, **Figure 6**). The high-risk group’s 1- and 2-year survival rates were 49.6% (95% CI: 38.7% to 63.4%) and 14.2% (95% CI: 6.8% to 29.7%), respectively, while the corresponding survival rates in the low-risk group were 100% (95% CI: 6.8% to 29.7%) and 90% (95% CI: 73.2% to 100%).

## 4 Discussion

The chemotherapy-immunotherapy combination is considered one of the most effective multimodal treatment strategies for ES-SCLC, which has demonstrated significant effectiveness. By blocking the immune escape pathways, this approach increases the immune system’s response to cancer cells (10). However, this translates into a long-term survival benefit only in a minority of patients, with a lack of clinical predictive models developed for immunotherapy era in ES-SCLC. The limitations of the historical approach in assessing the efficacy of chemotherapy in terms of anti-tumor activity and overall survival as well as its inability to take into account the interactions and responses generated by immunotherapy have been widely acknowledged (11). Therefore, it is essential to develop more efficient predictive models to identify potentially promising candidates for chemo-immunotherapy combinations. By including possible biomarkers and combining clinical indicators like patient demographics and tumor features, these models can improve patient outcomes. Among the potential predictors that were considered in the present predictive model for ES-SCLC patients receiving first-line chemo-immunotherapy, several factors were revealed to be significant in terms of outcomes of the treatment. These variables include older people, ECOG PS, brain metastases, hepatic metastasis, NLR, and immunotherapy cycles.

Earlier investigations have shown the impact of these parameters on the treatment response and overall prognosis in ES-SCLC patients. For instance, elderly patients have lower rates of OS, most likely due to co-morbidities and treatment tolerance. Our study suggests a shorter survival for older people, corroborating the results from prior research (12, 13). Moreover, Longo et al. established an association between increased age and poor prognosis disease, although this was not confirmed by multivariate analysis (14). ECOG PS, referring to a measure of functional status, has been used as an independent predictive factor, with higher scores being associated with poorer outcomes. ECOG PS 1-2 patients had a medium-term survival rate of 68.1% and a long-term survival rate of 65.1%, while ECOG PS 3-4 patients had a medium-term survival rate of 18.1% and a long-term survival rate of 0% (15). Another study also demonstrated that patients with PS 2-3 showed significantly poorer OS compared to patients with PS 0-1 (16). Both the univariate and multivariate analyses found that PS was a significant prognostic factor; the present model showed a negative connection between PS score and OS.

The presence of brain as well as hepatic metastases is a hallmark of increased disease volume and reduced therapeutic efficacy. Individual research has demonstrated that liver and brain metastases are negative prognostic factors; however, these findings are limited to individuals treated with first-line systemic chemotherapy (15, 16). Moreover, there has not been a detailed investigation of the immunotherapy impacts on this component. The current study augmented this information and included it in the model to serve as a clinical point of reference.

It has been reported that inflammatory prognostic biomarkers have prognostic significance in a range of tumor types. Among NLR, PLR, SII, and other factors, only NLR was related to prognosis in this investigation. NLR represents the balance between the innate and adaptive immune systems. Among these, neutrophils release chemokines and cytokines, which are crucial for the advancement of cancer. In response to malignancy, lymphocytes can stimulate a cytotoxic immune response as well. It has been found that raised NLR is linked to poor prognosis after immunotherapy for ES-SCLC (17, 18), while a sharp decline in NLR is consistent with exceptional outcomes in patients. This study further confirms the role and value of NLR in immunotherapy.

In recent years, immunotherapy has made remarkable progress in cancer treatment, and the optimization of treatment strategies represents an important field of research (19). By understanding the patient’s immune status and tumor microenvironment, personalized treatment plans can be better developed, thus improving the effectiveness of immunotherapy (20). Previously, the duration of immunotherapy has primarily been determined based on expert judgment and estimation rather than a standardized prognostic model. The inclusion of immunotherapy cycles in the present predictive model represents a progression in mapping therapies for each patient. Accurately determining the optimal number of immunotherapy cycles is crucial for maximizing treatment efficacy and minimizing potential adverse effects. However, it is feasible to uncover studies that suggest a positive OS when many CT lines are used. There is a lack of information on the impact of the number of cycles of immunotherapy. By analyzing patient data, the results suggest that specific cohorts may benefit from extended immunotherapy regimens, while others may not derive such benefit in terms of long-term survival after a certain number of cycles. This nuanced approach allows for personalized treatment strategies that encompass both patient-specific characteristics and treatment duration, ultimately guiding clinical decision-making.

The model provides several potential benefits in the context of ES-SCLC. The present model incorporates multitudes of clinical variables, indicating a comprehensive method for predicting treatment outcomes. This aids physicians in evaluating the best suitable therapy and customizing treatment procedures. Furthermore, this model demonstrates a broad interest in ES-SCLC, particularly in the context of emerging guidelines of treatment involving first-line chemotherapy and immunotherapy. This model provides beneficial assistance for clinicians in improving therapeutic approaches by offering insights into the elements that influence treatment response in this unique scenario.

Based on our results, using the nomogram, patients with ED-SCLC who were treated with chemo-immunotherapy can be classified into good- and poor-responder groups. However, the role of immunotherapy in the different respond groups is not the purpose of our study. We cannot conclude that immunotherapy only add financial toxicity to poor-responder group of patients. Due to the limited number of patients included in this study, we cannot further evaluated the role of immunotherapy and obtain a sounded conclusion. So further study should be performed to evaluate the role of immunotherapy in the poor-responder group of patients and the role of other treatment modality such as thoracic radiation in this kind of patients also need to be studied.

However, it is important to emphasize some limitations that are related to this study model. First, this model was derived from historical data, and therefore, the information it provides may be susceptible to biases and other variables that might distort the actual situation. Further studies with more extensive patient data should be undertaken to validate the findings and improve the model. Moreover, it is essential to acknowledge that the data upon which the model was developed and evaluated was from a single-center population. Therefore, it would be necessary to conduct external validation to apply the model to different populations.

## 5 Conclusion

In conclusion, this model for immunotherapy combined with first-line chemo-therapy in ES-SCLC patients has potential as a method for predicting treatment results. These characteristics enhance the ability to evaluate the clinical situation and assist physicians in making more informed treatment choices. However, further investigation is needed to ascertain the dependability and efficacy of the proposed model to ensure its effectiveness in clinics and improve patient outcomes.

## Data Availability

The original contributions presented in the study are included in the article/**Supplementary Material**. Further inquiries can be directed to the corresponding author.
